# Supplementary material for: Hybrid Models and Biological Model Reduction with PyDSTool
Source: PLoS Comput Biol. 2012 Aug 9;8(8):e1002628. doi: 10.1371/journal.pcbi.1002628 (PMC3415397; doi:10.1371/journal.pcbi.1002628)
Supplement: Text S4 — Complete source code for the PyDSTool package (version 0.88.120504). Includes API documentation and help files linking to web pages. This file is identical to the current public release on Sourceforge.net. (ZIP) [file pcbi.1002628.s004.zip › PyDSTool/html/PyDSTool.Toolbox.optimizers.helpers-pysrc.html]

xml version="1.0" encoding="ascii"?


PyDSTool.Toolbox.optimizers.helpers


| Home | Trees | Indices | Help | PyDSTool |
| --- | --- | --- | --- | --- |

Package PyDSTool ::
Package Toolbox ::
Package optimizers ::
Package helpers

[hide private]

[frames] | no frames]

# Source Code for Package PyDSTool.Toolbox.optimizers.helpers

```
 1  """ 
 2  Helper functions 
 3   
 4  Fitting functions : 
 5    - Quadratic defines a quadratic cost 
 6   
 7  NB : the first dimension of the cost, gradient or hessian is the number of 
 8  points to fit, the second is the dimension of the point if there is one. 
 9  This leads to the fact that the gradient returns in fact the jacobian of 
10  the function. 
11   
12  Finite Difference functions : 
13    - ForwardFiniteDifferences 
14    - CenteredFiniteDifferences 
15    - also, versions with in-built caching of previous values 
16  """ 
17   
18  from quadratic import * 
19   
20  from finite_difference import * 
21   
22  helpers__all__ = ['Quadratic', 'FiniteDifferencesFunction', 
23                    'ForwardFiniteDifferences', 
24                    'CenteredFiniteDifferences', 'ForwardFiniteDifferencesCache'] 
25   
26  __all__ = helpers__all__ 
27
```

  


| Home | Trees | Indices | Help | PyDSTool |
| --- | --- | --- | --- | --- |

|  |  |
| --- | --- |
| Generated by Epydoc 3.0.1 on Fri May 4 15:24:11 2012 | http://epydoc.sourceforge.net |
